# Inhibition of SIRT1 Reactivates Silenced Cancer Genes without Loss of Promoter DNA Hypermethylation

**DOI:** 10.1371/journal.pgen.0020040

**Published:** 2006-03-31

**Authors:** Kevin Pruitt, Rebekah L Zinn, Joyce E Ohm, Kelly M McGarvey, Sung-Hae L Kang, D. Neil Watkins, James G Herman, Stephen B Baylin

**Affiliations:** 1 The Sidney Kimmel Comprehensive Cancer Center at Johns Hopkins, Baltimore, Maryland, United States of America; 2 The Graduate Program in Cellular and Molecular Medicine, The Johns Hopkins University School of Medicine, Baltimore, Maryland, United States of America; The Babraham Institute, United Kingdom

## Abstract

The class III histone deactylase (HDAC), SIRT1, has cancer relevance because it regulates lifespan in multiple organisms, down-regulates p53 function through deacetylation, and is linked to polycomb gene silencing in *Drosophila*. However, it has not been reported to mediate heterochromatin formation or heritable silencing for endogenous mammalian genes. Herein, we show that SIRT1 localizes to promoters of several aberrantly silenced tumor suppressor genes (TSGs) in which 5′ CpG islands are densely hypermethylated, but not to these same promoters in cell lines in which the promoters are not hypermethylated and the genes are expressed. Heretofore, only type I and II HDACs, through deactylation of lysines 9 and 14 of histone H3 (H3-K9 and H3-K14, respectively), had been tied to the above TSG silencing. However, inhibition of these enzymes alone fails to re-activate the genes unless DNA methylation is first inhibited. In contrast, inhibition of SIRT1 by pharmacologic, dominant negative, and siRNA (small interfering RNA)–mediated inhibition in breast and colon cancer cells causes increased H4-K16 and H3-K9 acetylation at endogenous promoters and gene re-expression despite full retention of promoter DNA hypermethylation. Furthermore, SIRT1 inhibition affects key phenotypic aspects of cancer cells. We thus have identified a new component of epigenetic TSG silencing that may potentially link some epigenetic changes associated with aging with those found in cancer, and provide new directions for therapeutically targeting these important genes for re-expression.

## Synopsis

The propensity for cancer to arise and progress is influenced not only by gene mutations (genetic abnormalities), but also by defects in gene expression programs that are inherited from one dividing cell to another. This change in the inheritance of gene expression patterns not associated with changes in the primary DNA sequence is referred to as an epigenetic abnormality. In virtually every form of cancer, tumor suppressor genes (TSGs) and candidate TSGs are epigenetically altered such that the ability of these genes to become activated and lead to production of the corresponding proteins is lost. This so-called gene “silencing” is often linked with abnormal accumulation of methyl groups to DNA (DNA methylation) in a region of the gene that controls its expression. The SIRT1 protein is an enzyme that can remove acetyl groups attached to specific amino acids in a number of different protein targets and thereby regulate gene silencing in yeast. However, in mammalian cells this has not been demonstrated. Here, the authors show SIRT1 is involved in epigenetic silencing of DNA-hypermethylated TSGs in cancer cells. Inhibition of SIRT1 by multiple approaches leads to TSG re-expression and a block in tumor-causing networks of cell signaling that are activated by loss of the TSGs in a wide range of cancers. This finding has important ramifications for the biology of cancer in terms of what maintains abnormal gene silencing. Furthermore, the authors propose that their observations may have potential clinical relevance in suggesting new means for restoring expression of abnormally silenced genes in cancer.

## Introduction

A growing list of tumor suppressor genes (TSGs) and candidate TSGs are epigenetically silenced in virtually every cancer type, and this silencing has been associated with aberrant promoter DNA methylation [1–3]. In previous studies, silencing of these genes was shown to involve dense hypermethylation of 5′ CpG islands and hypoacetylation of lysine 9 and 14 on histone H3 (H3-K9 and H3-K14, respectively) [4,5]. Moreover, synergistic reactivation of these TSGs can be achieved only when class I/II histone deactylase (HDAC) inhibitors (HDIs) are employed to treat tumor cells after DNA demethylating agents, such as 5-deoxy-azacytidine (DAC), have first induced at least partial promoter demethylation [5,6]. This suggested a dominance of the DNA methylation over H3-K9/K14 deactylation for maintenance of the gene silencing [1]

Another important class of HDACs, the NAD^+^-dependent sirtuins, or class III HDACs [7], has recently received much attention. The most prominent human family member, SIRT1 (Q96EB6), has only been shown to regulate transcriptional repression of mammalian target genes that are either already basally expressed [8] or to regulate transcriptional repression of an integrated Gal4-fusion reporter plasmid [9–11]. Thus far, SIRT1 has not been linked to heterochromatin maintenance or heritable silencing of TSGs, nor has it been well studied for endogenous mammalian genes. The sirtuins have distinct specific inhibitors [12–14] and are not responsive to drugs like trichostatin-A (TSA) or other class I and II HDIs previously used to study promoter-hypermethylated TSGs. At least eight different class I/II HDIs are advancing in different phases of clinical trials for cancer treatment [15,16], but inhibitors of sirtuin deacetylases have not been investigated for such use. The human class III HDAC, SIRT1, already has cancer relevance because it regulates gene silencing and/or lifespan in multiple organisms [17–20], regulates p53 function [21–23], and plays a critical role in stress signaling [24,25]. In addition, the yeast SIRT1 ortholog, Sir2, directly mediates histone-dependent gene silencing [7,26], and its counterpart in *Drosophila* has been linked to polycomb gene silencing [27]*.* However, SIRT1 has not been demonstrated to mediate heritable silencing for endogenous mammalian genes.

## Results

**Figure 1:**
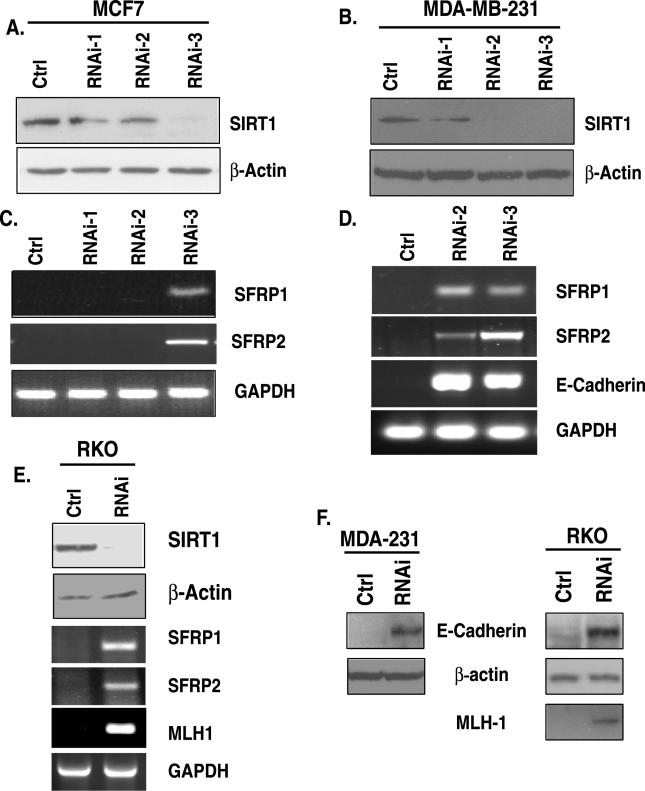
siRNA Knockdown of SIRT1 Causes Re-Expression of Epigenetically Silenced TSGs (A) RNAi-3 is most effective for reduction of SIRT1 in MCF7 cells. Retroviral expression vectors encoding SIRT1 cDNA that produce short hairpin loop RNA targeting either distinct regions of SIRT1 mRNA (RNAi-1, −2, or −3) or a control (ctrl) were used to infect MCF7. Western blot analysis for SIRT1 and β-actin was performed 48 h after two rounds of infection. (B) Both RNAi-2 and −3 are effective for reduction of SIRT1 protein in MDA-MB-231 cells as described in (A). (C) SIRT1 inhibition leads to TSG re-expression in MCF7 cells. RNA was isolated from parallel samples analyzed in (A), and RT-PCR was performed with intron-spanning primers specific for the genes *SFRP1* and *SFRP2*. GAPDH was also analyzed as a control. Only the shRNA (RNAi-3) that caused substantial reduction in SIRT1 protein leads to gene re-expression. Control samples in which no reverse transcriptase was added were analyzed separately, and all were negative for amplification of the indicated genes. (D) SIRT1 inhibition leads to TSG re-expression in MDA-MB-231 cells. RT-PCR was performed for analysis of the genes *SFRP1, SFRP2,* and *E-cadherin* as described in (A). Only the shRNAs (RNAi-2 and −3) that caused substantial reduction in SIRT1 protein lead to gene re-expression (E) SIRT1 inhibition leads to TSG re-expression in RKO cells. SIRT1 protein reduction by RNAi-3 (top panel) as described in (A) leads to gene re-expression of *SFRP1, SFRP2,* and *MLH1* as described in (C) (F) MDA-MB-231 and RKO cells infected with control or RNAi-3 shRNA as described in (A) were selected with puromycin for 3 d, and pooled colonies were harvested for Western blot analysis of protein re-expression that corresponded with the gene reactivation described in (D) and (E).

To determine whether SIRT1 specifically plays a role in silencing TSGs whose promoters have 5′ CpG islands that are densely hypermethylated, we first applied screens using RNA-interference (RNAi) to disrupt the function of this protein and evaluate the effects on the targets. Both breast and colon cancer cell lines were chosen for our study, and several RNAi sequences targeting SIRT1 specifically were tested for their efficacy. SIRT1 protein levels in both MCF7 (Figure 1A) and MDA-MB-231 (Figure 1B) breast cancer cells were reduced via retroviral infection with a pSuper-retro-RNAi construct encoding short hairpin loop RNA (shRNA) specific for “knocking down” SIRT1. Three RNAi constructs were tested, and the sequence termed RNAi-3 yielded the greatest knockdown in MCF7 (Figure 1A), whereas both RNAi-2 and RNAi-3 were very effective in reducing protein levels in MDA-MB-231 cells (Figure 1B). Since we infected cells with equivalent titers of virus encoding the shRNAs, we are not sure why RNAi-3 was the most effective, but as shown below, the degree of knockdown served as a good control since it correlates very well with effects on gene re-expression.

Strikingly, and correlating with the knockdown pattern of SIRT1 in each cell type, we observed re-expression of key TSGs that are frequently epigentically silenced in a number of different cancers. The anti-tumor genes identified all have promoter DNA hypermethylation, and they have important anti-tumor functions ranging from mediating proper epithelial cell differentiation to promoting cell–cell adhesion. The genes include members of the family of secreted frizzled-related proteins *(SFRP1* and *SFRP2),* which are frequently epigenetically inactivated during colon and breast cancer progression, and contribute to aberrant activation of Wnt signaling (Figure 1C and 1D) [6,28]. Additionally, SIRT1 was found to maintain silencing of *E-cadherin,* a gene mediating cell–cell adhesion that is also inactivated epigenetically in many cancers (Figure 1D) [29–31]. Finally, SIRT1 protein levels were also reduced in RKO colon cancer cells and SIRT1was found to maintain silencing of TSGs including the mismatch repair gene, *MLH1* (Figure 1E), for which epigenetic silencing and loss of function produces the microsatellite instability (MIN+) colon cancer phenotype [32,33] *.* Additionally, we found that the transcription factors encoding *GATA-4* and *GATA-5* genes, whose promoter DNA is hypermethylated [34], were also re-expressed in both colon and breast cancer cells (unpublished data).

To further determine whether the gene re-expression with this very specific approach for SIRT1 inhibition leads to protein re-expression, we performed parallel Western blots on samples for which proven antibodies are available. Consistent with gene re-expression, we found restoration of E-cadherin protein in breast and colon cancer cell lines and MLH1 in colon cancer lines in which these genes are hypermethylated and silenced (Figure 1F). These findings further demonstrate that SIRT1 specifically, and substantially, contributes to the aberrant heritable silencing of our panel of TSGs. Moreover, the levels of gene expression when SIRT1 function is reduced is similar to that observed for these genes when moderate doses of 5′-aza-deoxycytidine (Aza) is employed to achieve promoter demethylation [32,35]. Furthermore, we have demonstrated previously that the degree of protein re-expression for MLH1 obtained correlates with restored protein function in RKO cells [32].

**Figure 2:**
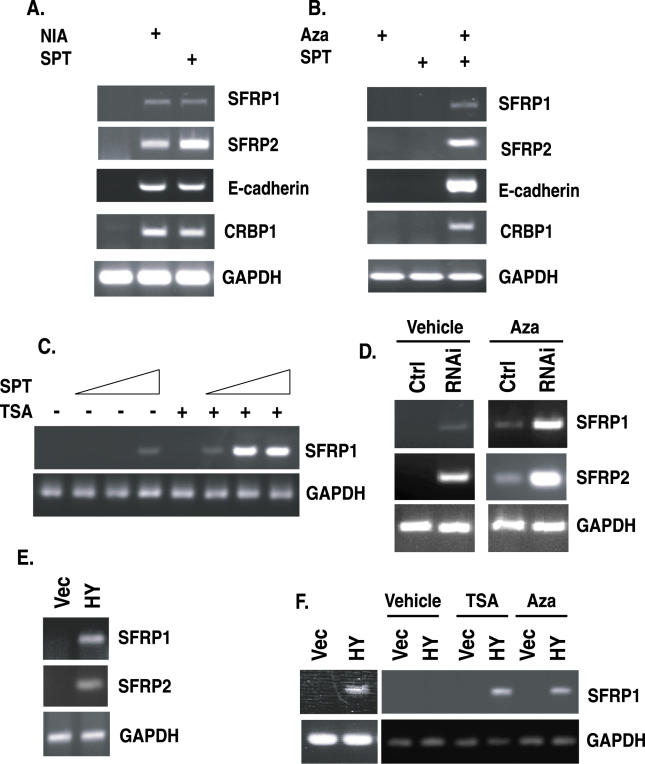
Pharmacologic and Dominant Negative Inhibition of SIRT1 Cause Re-Expression of TSGs and Synergize with 5-Deoxy-Azacytidine or TSA (A) Pharmacologic inhibition of SIRT1 causes TSG re-expression. MDA-MB-231 cells were treated with 15 mM NIA or 300 μM SPT for 21 h, RNA was isolated, and RT-PCR was performed with intron−spanning primers specific for the indicated genes. Control samples in which no reverse transcriptase was added were analyzed separately, and all were negative for amplification of the indicated genes. (B) Combined treatment with low doses of Aza and SPT synergizes in the re-expression of TSGs. MDA-MB-231 cells were treated with either 50 nM Aza (+), 100 μM SPT (+) or with both Aza and SPT (++), and 34 h later, RT-PCR was performed for the indicated genes as described in (A). (C) Combined treatment with SPT and TSA synergize in the re-expression of genes. MDA-MB-231 cells were treated with either 0, 50, 100, or 120 μM SPT alone for 34 h, or the treatment was followed by treatment with 300 nM TSA for 3 h prior to RNA isolation and RT-PCR analysis. (D) SIRT1 protein knockdown synergizes with low doses of Aza for gene re-expression. MDA-MB-231 cells were infected with low titers of virus for shRNA specific for SIRT1. Aza (100 nM) was added 24 h prior to RNA isolation, and RT-PCR analysis was performed for the genes *SFRP1, SFRP2,* and *GAPDH* as described in (A). (E) Dominant negative inhibition of SIRT1 leads to TSG re-expression in MCF7 cells. MCF7 cells were infected with virus encoding either pBabe (vec) or the catalytically inactive SIRT1H363Y (HY) mutant, and RT-PCR was performed as described in (A). (F) Dominant negative inhibition of SIRT1 leads to TSG re-expression and synergizes with TSA and Aza. As shown in the left panel, MDA-MB-231 cells were infected with a control (vec) or mutant SIRT1 virus (HY), and RT-PCR was performed as described in (A). MDA-MB-231 cells were infected with low titers of pBabe or pBabe-SIRT1H363Y retrovirus and subsequently treated with 100 nM Aza for 24 h or with 300 nM TSA for 3 h prior to harvest, and RT-PCR was performed.

To further assess the role SIRT1 plays in silencing TSGs whose promoter DNA is hypermethylated, we used two additional approaches. We applied a pharmacologic approach using the general sirtuin inhibitor, nicotinamide (NIA) [12,36], and the more sir2-specific inhibitor, splitomicin (SPT) [13,37]. Consistent with our above RNAi data, we found that these sirtuin inhibitors could cause the re-expression of the epigenetically silenced, hypermethylated TSGs studied above, and another such gene, *CRBP1,* in the human breast cancer cell lines MDA-MB-231 (Figure 2) or MCF7 (unpublished data). Using yet a third approach to assess the role that SIRT1 plays, we expressed a catalytically inactive, dominant negative inhibitor of SIRT1, SIRT1H363Y [21], and screened representative genes to further validate the specific involvement of this protein in repression of our panel of hypermethylated TSGs. In both MCF7 and MDA-MB-231 breast cancer cells in which SIRT1H363Y was expressed through retroviral infection, we observed a re-expression of *SFRP1*and *SFRP2* (Figure 2E and 2F [left panel]). Additionally, we saw the same effect for *GATA-4* in HCT116 colon cancer cells when the H363Y mutant was expressed, but not the wild type (unpublished data).

As discussed earlier, we have demonstrated previously that DNA methylation and histone deacetylation, involving class I and II HDACs, act as synergistic layers for TSG silencing in cancer and that inhibition of DNA methylation is dominant relative to the inhibition of deacetylation [6]. Thus, we also wanted to determine whether disruption of sirtuin function could collaborate with either inhibitors of DNA methylation or class I/II HDIs in TSG re-expression. In this regard, low doses of Aza (50 nM) or SPT (50 μM) that were ineffective as single agents could be combined to achieve synergistic re-expression of our gene panel as shown by representative genes in Figure 2B.

Strikingly, we also found synergy in gene activation by combining the class I/II HDI, TSA, with increasing doses of SPT to reactivate genes whose promoters have hypermethylated DNA (Figure 2C and unpublished data). To again assess the synergy with DNA demethylation, we used low titers of shRNA retrovirus and low-dose Aza, and observed a synergistic re-expression of *SFRP1* and *SFRP2* (Figure 2D). The specific contribution of SIRT1 inhibition to the synergistic effects of combining either Aza treatment or TSA with sirtuin inhibition was investigated using low titers of SIRT1H363Y retrovirus. We also observed the synergistic reactivation of *SFRP1* (Figure 2F, right panel), and *GATA-5* and *SFRP2* (unpublished data) in response to inhibition with the SIRT1 dominant negative SIRT1H363Y (HY) when used in low titers and combined with either Aza or TSA. These results provide strong evidence that, although SIRT1 inhibition alone is sufficient for the reactivation of our panel of TSGs, inhibition of DNA methylation and class I/II HDACs can cooperate with SIRT1 inhibition in such reactivation.

**Figure 3:**
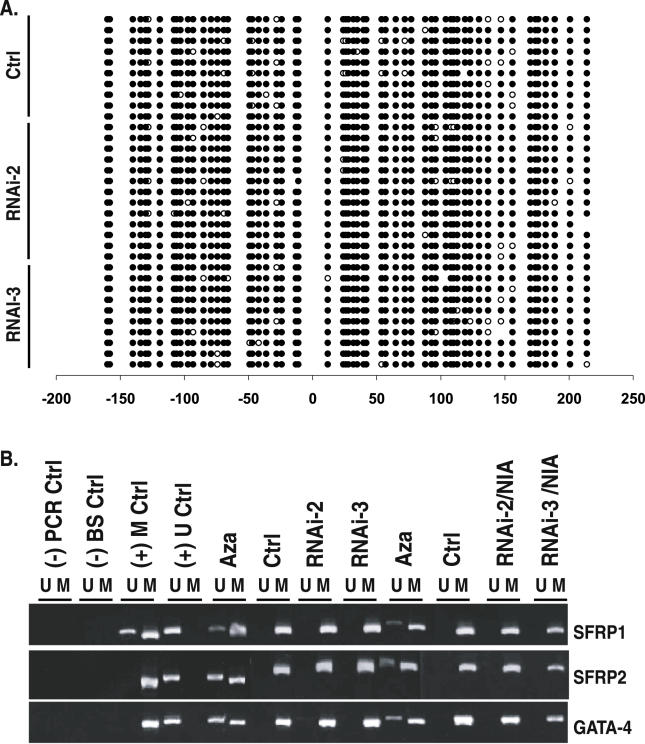
SIRT1 Inhibition Causes TSG Re-Expression without Changing Promoter DNA Hypermethylation (A) TSG re-expression occurs without changes in the methylation profile of multiple clones analyzed for *SFRP1* promoter methylation. Parallel samples analyzed in Figure 1D were subjected to bisulfite sequencing of the *SFRP1* promoter from MDA-MB-231 cells stably infected with control vector or RNAi-2 or RNAi-3 retrovirus. Open circles indicate unmethylated cytosines, and closed circles indicate methylated cytosines. Numbers at the bottom show the position of cytosines relative to the transcription start site, which is at position 0, and those with a minus sign (−) are upstream from this start site. The region sequenced encompasses the CpG island in which methylation status correlates with gene expression status. (B) MSP analyses of DNA from MDA-MB-231 cells stably expressing vector control, RNAi-2, or RNAi-3 retrovirus. From left to right: (-) PCR Ctrl indicates H_2_O only; (-) BS ctrl indicates bisulfite-treated H_2_0; (+) M ctrl indicates the cell line in which *SFRP1* is partially methylated and *SFRP2* and *GATA4* are fully methylated; and (+) U ctrl indicates the Tera-2 cell line in which each gene is unmethylated. All remaining lanes are for MDA-MB-231. From left to right: Aza indicates 1 μM Aza (24 h) treatment; Ctrl indicates empty vector infection; RNAi-2 indicates shRNA-2 infection alone; RNAi-3 indicates shRNA-3 infection alone; Aza indicates 1 μM Aza (24 h) treatment of control cells; Ctrl indicates empty vector infection + vehicle; RNAi-2 indicates shRNA-2 infection + 5 mM NIA treatment; and RNAi-3 indicates shRNA-3 infection + 5 mM NIA treatment.

Given that SIRT1 appears to be intimately involved in maintaining silencing of the genes under study whose promoter DNA is densely hypermethylated, we wanted to determine whether the mechanism of reactivation coincided with any changes in the DNA methylation status at the re-expressed TSG promoters. To assess this, we performed extensive bisulfite sequencing of samples in which TSGs were reactivated by transient knockdown of SIRT1 by RNAi as shown in Figure 1 and by stable knockdown of SIRT1. Strikingly, we observed no change in promoter methylation of *SFRP1* or *GATA-5* (Figures 3A, S1, and S2). Moreover, a very sensitive, methylation-specific PCR (MSP) approach for detection of methylation status [38] yielded identical results (Figure 3B) to those from bisulfite sequencing. In all previous studies of these genes, a similar degree of reactivation with Aza is always accompanied by significant promoter demethylation as assessed by MSP analyses or bisulfite sequencing [28,34]. Furthermore, when the cells with stable RNAi knockdown were treated with NIA to further inhibit any remaining SIRT1 protein, as shown in the RNAi-2/NIA and RNAi-3/NIA lanes in Figure 3B, we observed no restoration to the unmethylated state for genes examined, even though they were re-expressed. Thus, it appears that SIRT1 inhibition alone is sufficient for the reactivation of tested TSGs even when dense promoter DNA methylation is maintained.

**Figure 4:**
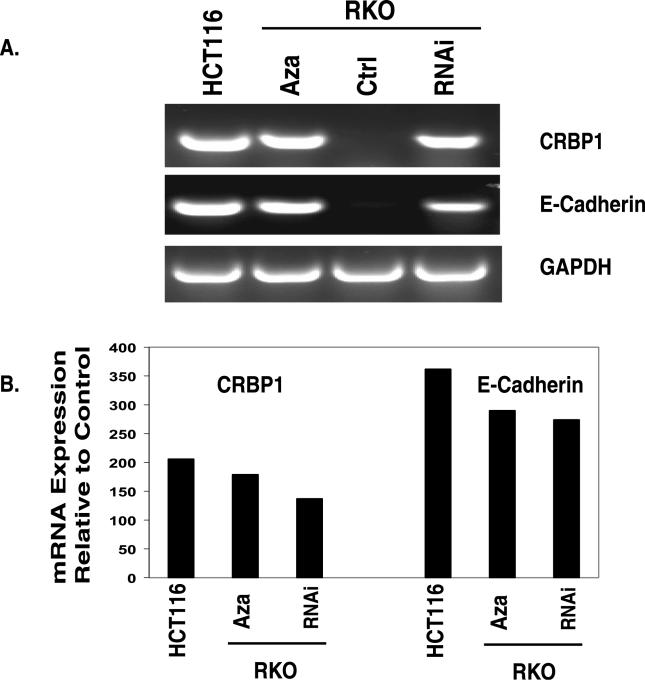
SIRT1 Inhibition Causes Re-Expression of Epigenetically Silenced TSGs (A) RKO cells were infected and stably selected to express short hairpin loop RNA targeting either a region unique to SIRT1 mRNA or a control (ctrl). To inhibit any residual SIRT1 protein, remaining RNAi-expressing cells were treated with 700 μM SPT and control samples were treated with DMSO for 24 h. For comparison, control RNA was isolated from parallel samples from HCT116 cells in which the two genes under study, *CRB1* and *E-cadherin,* do not have promoter DNA hypermethylation and are basally expressed. RKO cells were also treated with 0.5 μM Aza (24 h), and samples were analyzed as described in Figure 1A; RT-PCR was performed with intron-spanning primers specific for the two genes. GAPDH was also analyzed as a control. Only the shRNA (RNAi-3) that caused substantial reduction in SIRT1 protein leads to gene re-expression. Control samples in which no reverse transcriptase was added were analyzed separately, and all were negative for amplification of the indicated genes. (B) Parallel samples described above were analyzed using real-time quantitative PCR. The level of TSG re-expression induced by Aza treatment or SIRT1 inhibition as described in (A) was compared to levels of expression in HCT116 cells in which the TSGs are basally expressed.

One question that emerges with the above re-expression of genes induced by SIRT1 reduction in the face of retained DNA methylation is how the extent of transcription achieved compares to expression of these genes when DNA methylation alone is markedly reduced or absent. To examine this, we compared by RT-PCR (Figure 4A) and by quantitative real-time RT-PCR (Figure 4B) the re-expression achieved by SIRT1 knockdown of two genes with the basal expression of these same genes in an another cancer cell line in which the promoter DNA is not hypermethylated (Figure 4). In RKO cells in which SIRT1 protein levels were reduced via shRNA, and the residual SIRT1 protein was inhibited with SPT, we observed a restoration of CRBP1 and *E-cadherin* mRNA transcripts to about 60%–75% of the levels for their basal expression in HCT116 cells in which the promoter DNA is not hypermethylated. Similarly, levels of re-expression of the genes after SIRT1 reduction were comparable to those achieved after decreased DNA methylation using intermediate doses of Aza (500 nM) (Figure 4). These results provide evidence that SIRT1 inhibition plays a significant role in TSG re-expression even when promoter DNA methylation is retained and that SIRT1 likely cooperates with factors other than DNA methylation to help mediate the gene silencing.

**Figure 5:**
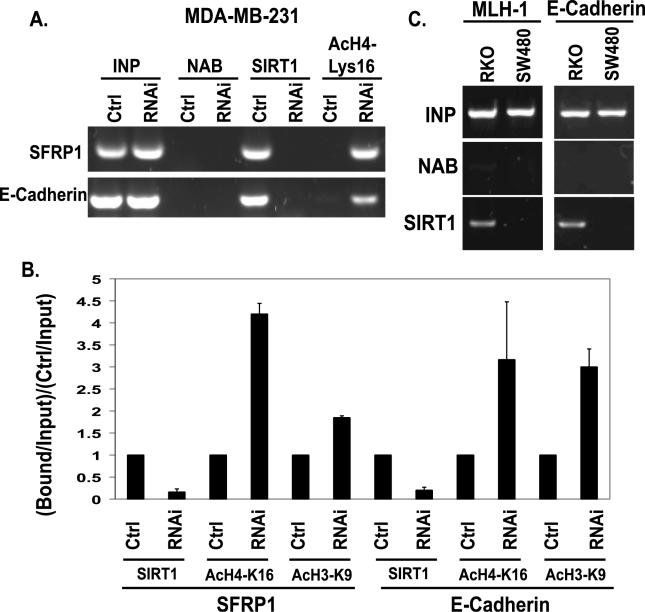
SIRT1 Inhibition Causes Increases in Histone H4-K16 Acetylation at the Promoter of Re-Expressed Genes (A) Pooled populations of MDA-MB-231 cells stably selected to express RNAi constructs were analyzed via ChIP. These samples were isolated in parallel to those analyzed in Figure 3B. ChIP was performed with antibodies against SIRT1, acetylated histone H4, lysine 16 (H4-K16), or with no antibody (NAB) controls. Each promoter sequence was amplified by PCR under linear conditions for the genes *SFRP1* and *E-cadherin*. (B) The average change in SIRT1 localization, acetylation of H4-K16, and acetylation of H3K9 at the *SFRP1* and *E-cadherin* promoters as measured by ChIP was quantitated for multiple experiments. Error bars indicate the standard deviation for multiple experiments. (C) SIRT1 localizes to the promoters of silent genes whose DNA is hypermethylated, but not to these same promoters in cells in which the genes are expressed. ChIP was performed with antibodies against SIRT1 in RKO and SW480 colon cancer cells. As shown in the left panel, SIRT1 localizes to the *MLH1* promoter in RKO cells in which the gene is silent, but not to the *MLH1* promoter in SW480 cells in which it is expressed. As shown in the right panel, SIRT1 localizes to the *E-cadherin* promoter in RKO cells in which the gene is silent, but not to the *E-cadherin* promoter in SW480 cells where it is expressed.

How does SIRT1 function in contributing to the silencing of DNA hypermethylated TSGs? To address this question, we examined whether SIRT1 localizes to the promoters of the hypermethylated genes studied and directly modulates histone changes. We performed chromatin immunoprecipitation (ChIP) assays in MDA-MB-231 cells and observed SIRT1 localization at DNA-hypermethylated and silenced promoters for *SFRP1, E-cadherin,* and *GATA-5* (Figure 5 and unpublished data) and at the silenced *MLH1* and *E-cadherin* promoters in RKO colon cancer cells (Figure 5C). This localization was reduced with shRNA knockdown of SIRT1 (Figure 5A). Importantly, SIRT1 was absent from the promoters of the genes such as *MLH1* and *E-cadherin* when their promoter DNA is not hypermethylated and the genes are basally expressed in the SW480 colon cancer cells (Figure 5C).

We next determined how modifications of lysine residues known to be associated with transcriptional repression mapped with SIRT1-associated gene silencing. During *SFRP1* reactivation, and concurrent with shRNA knockdown of SIRT1, we observed robust increases in acetylation of H4-K16 (Figure 5A and 5B) which has been documented as a direct target of SIR2 in yeast [39–41] and a preferential target in human cells for an introduced SIRT1 induction reporter system [11]. Additionally, we observed significant increases in the levels of H4-K16 acetylation at the *SFRP1, E-cadherin,* and *GATA-5* promoters (Figure 5A and 5B, and unpublished data). We observed modest increases in H3-K9 acetylation at the *SFRP1* promoter and more substantial increases in H3-K9 acetylation at the *E-cadherin* promoter (Figure 4B). This latter modification has been tied to control by both class I and II HDACs, and SIRT1 [7,42].

**Figure 6:**
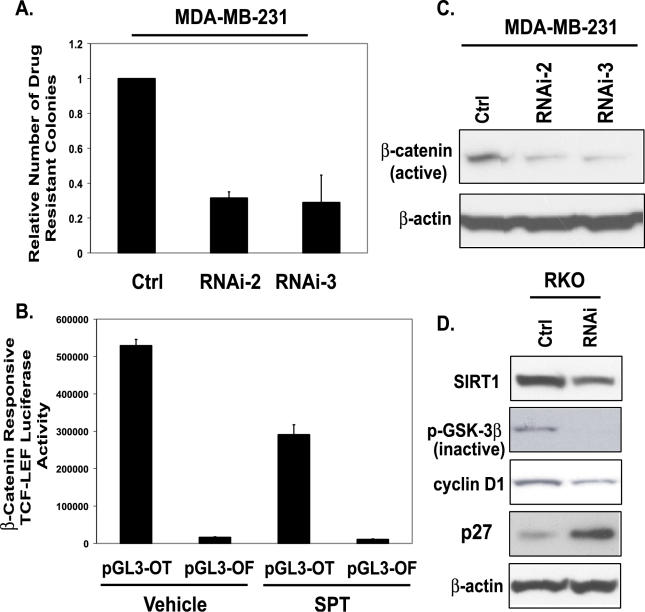
SIRT1 Inhibition Affects Key Phenotypic Aspects of Cancer Cells (A) MDA-MB-231 cells were infected for two rounds with RNAi-2 and −3 retrovirus, and puromycin-resistant colonies were counted after 3 d of selection. Error bars indicate standard deviation from the average of three experiments. (B) RKO cells were transfected with 500 ng of pGL3-OT, a TCF-LEF−responsive reporter, or pGL3-OF, a negative control with a mutated TCF-LEF binding site in combination with 10 ng of pRL-CMV vector. Twenty-four hours post-transfection, cells were treated with either vehicle (DMSO) control or with 700 μM SPT for 24 h. *Firefly* luciferase activity was measured and normalized to the *Renilla* luciferase activities. (C) As described in (A), pooled populations of MDA-MB-231 cells stably expressing RNAi-2 or RNAi-3 were harvested, protein concentrations were determined, and Western blot analysis was performed. An antibody that specifically recognizes the unphosphorylated (active) form of β-catenin was used, and on the same blot, β-actin was probed to ensure equal loading. (D) Western blot analysis was performed on RKO cells expressing control or SIRT1 RNAi. Antibodies against SIRT1, phospho-GSK3β (inactive), cyclin D1, p27, and β-actin were used for Western blotting. On the same blot, β-actin was probed to ensure equal loading.

Finally, from an overall cellular phenotype, we might predict that, if SIRT1 is involved in the repression of TSGs, inhibiting its function and concomitant re-expression of such genes should affect cell growth and/or viability. The numbers of DNA-hypermethylated and silenced TSGs in the cancer cell lines under examination make a direct analysis of this difficult. However, we tested the effects of SIRT1 on a series of colon and breast cancer phenotypic characteristics that would be predicted to change dramatically with re-expression of the TSGs under study. First, we examined the numbers of drug-resistant colonies that are formed during drug selection of cells for stable siRNA (small interfering RNA) knockdown of SIRT1. As shown in Figure 6A, we observed a sharp reduction in cell colonies during such selection.

Although the re-expression of many genes could account for the type of phenotypic change shown above, we queried whether reactivation of *SFRP* genes might be involved. We have shown previously that the silencing of the *SFRP1* and -*2* genes is important for aberrant activation of the Wnt pathway in colon cancer cells, and their re-introduction into such cells in which the genes are silenced causes sharp down-regulation of Wnt pathway function and apoptosis. [28]. First, we tested for the possible impact of the re-expression of these genes in colon cancer cells by examining key parameters of the Wnt signaling pathway following SIRT1 inhibition. We found a 50% reduction in the activation of a β-catenin–responsive TCF reporter construct, a canonical readout for Wnt pathway activity in colon cancer cells [28,43,44] with SPT treatment of RKO colon cancer cells (Figure 5B). Additionally, we found a 50% reduction in the activation of a β-catenin–responsive *cyclin-D1* promoter reporter construct [45,46] with SPT treatment of RKO cells (data not shown). We also observed suppression of other Wnt pathway signaling parameters in that there was a decrease in inactive phospho-GSK-3β, a member of the β-catenin destruction complex, and a reduction in cyclin D1 levels, a downstream target of nuclear β-catenin (Figure 6). We further observed that inhibition of SIRT1 lead to increases in p27 protein levels in RKO cells, an observation consistent with another report [47] using dominant negative inhibition of SIRT1 in another cell type. As demonstrated in Figures 1 and 2 in breast cancer cells, SIRT1 is involved in the silencing of SFRP1 and −2. Moreover, MDA-MB-231 cells express the wnt7b oncogene [48]. In MDA-MB-231 cells in which SIRT1 was inhibited stably by RNAi, we observed a sharp reduction in the levels of unphosphorylated or active β-catenin (Figure 5B). Thus, SIRT1 inhibition causes re-expression of *SFRP*s that antagonize WNT signaling. Furthermore, SIRT1 inhibition causes re-expression of the *E-cadherin* gene, whose protein product complexes with β-catenin, and this gene reactivation collectively may suppress the constitutive activation of the WNT signaling pathway.

## Discussion

Our findings for a prominent role for SIRT1 in epigenetic silencing of genes whose promoters are hypermethylated in cancer cells has important ramifications for the biology of cancer. Multiple actions now attributed to SIRT1 indicate that this protein could have important oncogenic roles. The effects of knocking down SIRT1 levels on cancer cell phenotypic features as found in our present study attest to this. The mechanisms by which SIRT1 could serve as an oncogene are multifactorial. First, there are data suggesting high expression of this protein in a number of cancer cell lines [49,50], and we have observed this as well (unpublished data and [51]). Second, SIRT1 deacetylates several transcription factors that could potentially compromise TSG function, such as for p53 [21–23]. Third, single-copy increases in SIRT1 orthologs in multiple organisms or application of sirtuin activators have been shown to prolong cellular lifespan [7,52]. Although this may obviously have beneficial effects in multiple cell settings, such prolongation may have a damaging effect from the standpoint of selecting for neoplastic cells during tumor progression.

We now provide another mechanism through which SIRT1 might prolong survival of cells at risk for transformation by participating in abnormal epigenetic silencing of TSGs. One possible scenario that ties this role of SIRT1 to a cascade of epigenetic events observed in cancer comes from our recent observation that *HIC1,* a gene that is frequently epigenetically silenced early in tumorigenesis, can be localized to the *SIRT1* promoter. Thus, in Hic1-null MEFs (mouse embryonic fibroblasts), there is a corresponding increase in SIRT1 levels [51]. Our current results indicate that the role of increased levels of SIRT1 in the silencing of additional TSGs could also contribute to its oncogenic potential and suggests a series of epigenetic feedback events that would all predispose cells to aberrant gene silencing. We tested the effects of SIRT1 on a series of colon and breast cancer phenotypic characteristics that would be predicted to change dramatically with re-expression of the TSGs under study. We observed a reduction in the numbers of drug-resistant colonies that are formed during drug selection of cells for stable siRNA knockdown of SIRT1. Although we observed a sharp reduction in cell colonies during such selection, we acknowledge that there is no way to know to what degree this effect is solely mediated by the role of SIRT1 in the gene silencing studied in our current work and/or how this would co-operate with other SIRT1-mediated events. However, this result is consistent with another report demonstrating that SIRT1 reduction via RNAi induces either growth arrest or apoptosis in human epithelial cancer cells and suggests an oncogenic role for increased levels of this protein in cancer cells [53].

Another important possibility from our findings also relates to the role of SIRT1 in lifespan prolongation and delay of aging effects. The process of aging has been tightly linked to increasing promoter DNA hypermethylation in cancer-prone sites such as the human colon [54]. Certainly it will now be important to study further whether this aging response may involve related increases in SIRT1 levels that may, in turn, facilitate aberrant gene silencing. From our observations it is clear that SIRT1 is necessary for maintaining aberrant silencing of TSGs, but the question still remains whether its increased expression is sufficient for the initiation of TSG silencing.

The mechanism through which SIRT1 participates in the gene silencing accompanying DNA hypermethylation of TSGs is also important to understand. Our present data indicate that the protein plays its role by localizing to the promoters of such silenced genes and deacetylating key histone lysine residues that are known to be critical for transcriptional repression. The targeting events for this recruitment will additionally be important to understand. Interestingly, the recent report that cancer cells have increased overall levels of deacetylation of the known histone target of SIRT1, H4-K16 [55], could well be related to the findings we now report at localized regions of aberrantly silenced TSGs. The role of SIRT1 at other silenced genes, including those in normal cells is not known. Considering the observations described here, an important focus of future work should involve testing whether SIRT1 associates with those few DNA-methylated genes in normal settings that contain promoter CpG islands, such as those on the inactive X chromosome of females or silenced alleles of imprinted genes.

Finally, our findings have potential clinical relevance. Combination therapies involving DNA-demethylating agents and class I/II HDAC inhibitors are receiving much attention for their potential therapeutic use in restoring expression of abnormally silenced genes in cancer [15,16]. Targeting SIRT1 in these strategies may be especially important. We have shown that blocking SIRT1 function synergizes with both promoter demethylation and inhibition of class I and II HDACs for gene reactivation and associated chromatin modification changes. Moreover, this inhibition of SIRT1 leads to gene reactivation even with retention of DNA methylation. Our findings then suggest new directions for targeting reversal of abnormal gene silencing and demonstrate the importance of continued study, which may lead to the eventual translation into the clinic.

## Materials and Methods

### Cell culture and retroviral infection.

MDA-MB-231, MCF7, HCT116, SW480 RKO, and Phoenix cells (ATCC, Rockville, Maryland, United States) were cultured in Dulbecco's modified Eagle's medium supplemented with 10% fetal bovine serum and 1% penicillin/streptomycin (Invitrogen, Carlsbad, California, United States). Retroviral infection was performed using either single or multiple rounds of infection. Briefly, Phoenix cells were transfected with either pBabe, pBabe-SIRT1H363Y, pSUPERretro, pSUPERretro-SIRT1-RNAi-1–3 (NM_012238 positions 410, 589, and 1091; Oligo Engine, Seattle, Washington, United States) using Lipofectamine 2000 (Invitrogen). After 48 h of transfection, the medium containing retrovirus was collected, filtered, and supplemented with Polybrene prior to infection of target cells (MDA-MB-231, MCF7, or HCT116). Infected cells were either harvested 24–48 h later or subjected to selection with 2–3 μg/ml puromycin for a week prior to harvest and analysis.

### RNA and protein preparation and analysis.

Total RNA was extracted (Invitrogen) according to the manufacturer's instructions and subjected to reverse transcription followed by both quantitative real-time and semi-quantitative polymerase chain reaction. For quantitative real-time analyses, the QuantiTect SYBR Green PCR kit (Qiagen, Valencia, California, United States) was used and the amplification conditions consisted of an initial 10-min denaturation step at 95 °C, followed by 40 cycles of denaturation at 95 °C for 15 s and annealing and extension for 30 s and 60 s, respectively. A BioRad iCycler was used (BioRad, Hercules, California, United States), and for quantitation the comparative cycle threshold (Ct) method was used, normalizing the Ct values for the indicated gene to the Ct values of GAPDH relative to a control sample. For conventional PCR, at least two independent sets of intron-spanning primers [28,34,56] were used for the analysis of multiple genes, such as *CRBP1,* (NM_002899), *E-cadherin,* (L34545), *SFRP1,* (BC036503), *SFRP2,* (BC008666), and *Gata-4* (L34357). For Western blots, cells stably expressing RNAi constructs were harvested in 50 mM Tris-HCl, 1% NP-40, .25% sodium deoxycholate, 150 mM NaCl, 50 mM sodium fluoride, 1 mM dithiothreitol, 1 mM AEBSF, 1× Complete protease inhibitor cocktail (Roche, Basel, Switzerland). Protein concentrations were measured by BCA (Pierce Biotechnology, Rockford, Illinois, United States). Protein extracts were subjected to polyacrylamide gel electrophoresis using the 4%–12% NuPAGE gel system (Invitrogen), transferred to PVDF (Millipore, Billerica, Massachusetts, United States) membranes, and immunoblotted using antibodies that specifically recognize SIRT1 (DB083; Delta Biolabs, Gilroy, California, United States, and 05–707; Upstate, Charlottesville, Virginia, United States), E-cadherin (Transduction Laboratories 610182; BD Biosciences, San Diego, California, United States), hMLH1 (551091; BD Biosciences), cyclin D1 (556470; BD Biosciences), p27^Kip1^ (Transduction Laboratories K25020; BD Biosciences), the unphosphorylated (active) form of β-catenin (05–665; Upstate), and phospho-GSK3β (05–643; Upstate). On the same blot, β-actin (Sigma, St. Louis, Missouri, United States) was probed to ensure equal loading.

Reporter assays were performed as described previously using the b-catenin–responsive TCF reporter [28] and the cyclin D1 reporter. Briefly, prior to transfection, RKO cells were plated in six-well tissue culture dishes and grown until they reached 80%–90% confluence. Cells were transfected with 500 ng of pGL3-OT, a TCF-LEF−responsive reporter, or pGL3-OF, a negative control with a mutated TCF-LEF binding site in combination with 10 ng of pRL-CMV vector. Twenty-four hours post-transfection, cells were treated with either vehicle (DMSO) control or with 700 μM SPT for 24 h. According to the manufacturer's instructions, *Firefly* luciferase activity was measured via a luminometer (BD Biosciences) and normalized to the *Renilla* luciferase activities by using the Dual Luciferase Reporter System (Promega, Madison, Wisconsin, United States).

### ChIP.

ChIP analysis was performed as previously described [4] with a few modifications. Antibodies to SIRT1 (05–707 and 07–313), acetyl-sH3-K9 (07–352), and acetyl-H4-K16 (07–329) were obtained from Upstate. Antibodies to SIRT1 were also obtained from Delta Biolabs (DB083). Primers (Forward: AGCCGCGTCTGGTTCTAGT; Reverse: GGAGGCTGCAGGGCTG) were designed for the *SFRP1* promoter spanning −163 to +12 relative to the transcription start site (+1) and were amplified by PCR under linear conditions. Enrichment was calculated as the ratio between the net intensity of the bound *SFRP1* sample divided by the input and the vector control sample divided by the input. Primers for E-cadherin were (Forward: TAGAGGGTCACCGCGTCTATG) and (Reverse: GGGTGCGTGGCTGCAGCCAGG), which encompass a CAAT signal.

### MSP and bisulfite sequencing.

MSP and bisulfite sequencing were performed as previously described [28,38] on DNA from MDA-MB-231 cells both transiently and stably infected with control vector or RNAi retrovirus.

## Supporting Information

Figure S1 SIRT1 Inhibition Causes TSG Re-Expression without Changing *SFRP1* Promoter DNA Hypermethylation Bisulfite sequencing was performed on the *SFRP1* promoter from MDA-MB-231 cells stably infected with control vector or RNAi-3 retrovirus as described in Figure 3. Open circles indicate unmethylated cytosines and closed circles indicate methylated cytosines. Numbers at the bottom show position of cytosines relative to the transcription start site, which is at position 0, and those with a minus sign (−) are upstream from this start site. The region sequenced encompasses the CpG island in which methylation status correlates with gene expression status.
(2.0 MB EPS)

Figure S2 SIRT1 Inhibition Causes TSG Re-Expression without Changing *GATA-5* Promoter DNA Hypermethylation Bisulfite sequencing was performed on the *GATA-5* promoter from MDA-MB-231 cells stably infected with control vector or RNAi-3 retrovirus as described in Figure 3. The region sequenced has previously been shown to be the region in which methylation occurs that most closely correlates with transcriptional activity. Open circles indicate unmethylated cytosines, and closed circles indicate methylated cytosines. Numbers at the bottom show position of cytosines relative to the transcription start site, which is at position 0, and those with a minus sign (−) are upstream from this start site. The region sequenced encompasses the CpG island in which methylation status correlates with gene expression status.
(1.5 MB EPS)

### Accession Numbers

The National Center for Biotechnology Information (NCBI) (http://www.ncbi.nlm.nih.gov) accession numbers for the genes and gene products discussed in this paper are *CRBP1* (GeneID: 5947), *cyclin-D1* (GeneID: 595), *E-cadherin* (GeneID: 999), *GATA-5* (GeneID: 140628), *MLH1* (GeneID: 4292), *SFRP1* (GeneID: 6422), and *SFRP2* (GeneID: 6423). The accession numbers for the proteins discussed in this paper are β-catenin (P35222), cyclin D1 (P24385), phospho-GSK-3β (P49841), and Sir2 (P53685).

## Abbreviations

ChIP: chromatin immunoprecipitation
Ct: cycle threshold
HDAC: histone deactylase
HDI: histone deactylase inhibitor
MSP: methylation-specific PCR
NIA: nicotinamide
RNAi: RNA-interference
shRNA: short hairpin loop RNA
SPT: splitomicin
TSA: trichostatin-A
TSG: tumor suppressor gene
